# Does Relationship-Contingent Self-Esteem Play a Role in the Stress to Impaired Control Pathway to Alcohol-Related Problems in a College Student Sample?

**DOI:** 10.3390/bs13020185

**Published:** 2023-02-17

**Authors:** Elena Kalina, Krystina Boyd-Frenkel, Julie A. Patock-Peckham, Lauren Schneidewent, Matthew L. Broussard, Robert F. Leeman

**Affiliations:** 1 Department of Health Education and Behavior, University of Florida, 1864 Stadium Rd., Gainesville, FL 32611, USA; 2 Department of Psychology, UC Irvine, Irvine, CA 92617, USA; 3 Department of Psychology, Arizona State University, Psychology Building, Tempe, AZ 85287, USA; 4 Department of Psychology, Northern Arizona University, 1100 S Beaver St., Flagstaff, AZ 86011, USA; 5 Department of Health Sciences, Bouvé College of Health Sciences, Northeastern University, Boston, MA 02115, USA

**Keywords:** relationship-contingent self-esteem, impaired control over alcohol, sex differences, alcohol-related problems, evolutionary theory, stress

## Abstract

The Appraisal-Disruption Model (ADM) suggests that individuals use alcohol as a means of dampening negative self-talk. Relationship-contingent self-esteem (RCSE) emerges from validating one’s self-esteem depending on one’s romantic relationship(s) and is known to predict alcohol-related problems. We hypothesized that RCSE indirectly predicts drinking outcomes through the mediating mechanism(s) of stress and impaired control over alcohol (IC; drinking to excess beyond one’s own intentions). We fit a multiple-group structural equation model with self-report survey data from 479 college students. We used a 20,000 bootstrap technique to examine possible mediated pathways. Consistent with evolutionary theory, our model was moderated by sex: more variance in alcohol-related problems was explained for women (R^2^ = 0.479) than for men (R^2^ = 0.280). RCSE was directly linked to more stress. Furthermore, higher levels of RCSE were indirectly linked to more IC through increased stress, and in turn, more heavy episodic drinking and alcohol-related problems for both men and women. Consistent with the ADM, those with higher levels of RCSE experienced more stress and, in turn, more IC and subsequent adverse alcohol outcomes. Thus, therapists targeting alcohol use disorders (AUDs) may wish to determine if their client’s self-esteem changes dramatically based on their moment-to-moment appraisal of their intimate relationships.

## 1. Introduction

*“He was gone… Love, life, meaning… over”*

*Bella about Edward, New Moon* [1].

An evolutionary–biological framework suggests that women need to be far more careful in their mate selection than men [2]. When pre-existing relationships exist, women may go to greater lengths to maintain those romantic partners due to their greater investment in offspring [3]. This behavior can be motivated by relationship-contingent self-esteem (RCSE), an unhealthy form of self-concept that is fundamentally rooted in one’s romantic relationships [4]. RCSE emerges from validating one’s own worth based on a damaging appraisal of the events in one’s romantic relationship(s) [4] (p. 609); [5]. With contingent self-esteem, one is more likely to perceive criticisms, mistakes, and failures as threats [6]. In addition, high RCSE is associated with an over-concern with others’ perceptions, increased body shame, lower autonomy, increased anxiety in social situations, attachment anxiety, and negative emotions [4,7]. Ironically, those with higher RCSE may have lower relationship satisfaction and decreased feelings of closeness with their partner [4] (p. 623). These associations suggest that living with high RCSE insinuates a greater overall stressful life experience [8]. Thus, stress is likely a mechanism through which some individuals high in RCSE experience dysregulated drinking and related problems.

### 1.1. Appraisal-Disruption Model (ADM)

The Appraisal-Disruption Model (ADM) suggests that the pharmacology of alcohol disrupts the processing “of stressful information by constraining the spread of activation of associated information previously established in long-term memory” [9] (p. 459) [10]. According to the ADM, alcohol can disrupt unhappy thoughts or self-evaluations [11]. The ADM suggests that individuals use alcohol to dampen negative self-talk, thereby weakening stress and anxiety symptoms [9,10]. Consistent with the ADM, individuals who are both nondependent and dependent on alcohol report drinking alcohol to cope with chronic stress [12]. Relationships between global self-esteem and alcohol-related-problems are mediated by drinking to cope [13]. Relatedly, increased RCSE is associated with increased drinking to cope and alcohol-related problems [14]. In addition, increased RCSE interacted with less relationship satisfaction to predict stronger coping motives for drinking and consequently, increased alcohol-related problems among men [8]. Thus, there is evidence to suggest that alcohol may dampen negative self-talk from stress related to high RCSE, as the ADM suggests.

### 1.2. Stress and Alcohol Consumption

The current investigation expands upon existing evidence by testing an indirect relationship of RCSE to dysregulated drinking and related problems through stress. Consuming alcohol for its anxiolytic properties is a risk factor for alcohol use disorders (AUDs) [8,15]. Evidence suggests relationships between higher levels of anxiety and depression are related to increased drinking [16], specifically increased drinking days and drinks per occasion [17,18]. Furthermore, stress and stressful life experiences are linked to increases in drinking as a method to cope [19]. In terms of romantic stress, there are clear links between stressful interpersonal events and AUDs [20]. For instance, interpersonal conflict, as in negative romantic relationship events, has been associated with drinking to alleviate negative effects [21,22]. For adults with low trait self-esteem, negative romantic relationship events were associated with a higher desire to drink and increased daily drinking [23]. Evidence also suggests a direct link from lower global self-esteem to more stress, with stress mediating the association between self-esteem and alcohol-related-problems [24]. Additionally, it has been found that stress mediates the relationship between contingent self-esteem and alcohol-related problems [13].

### 1.3. Impaired Control, Heavy Episodic Drinking, and Alcohol-Related Problems

Impaired control over alcohol (IC) is defined as “a breakdown of an intention to limit consumption” [25] (p. 701). IC is characterized as drinking more drinks or for a longer time than intended [26] and has been regarded as impulsivity specific to the drinking context [27]. Greater IC is associated with adverse drinking outcomes such as alcohol use quantity, frequency, heavy episodic drinking (4+ drinks for women, 5+ drinks for men) [28], and alcohol-related problems in young adults [27,29,30,31]. Alcohol-related problems are moderate problems with alcohol use [32] that are directly associated with IC [33]. Links between IC and alcohol outcomes are well-established, but in contrast, links between stress and IC are less explored. However, a direct link from stress to IC has been established with both survey techniques examining past month stress [34] and causal experimental investigations examining acute stress manipulations [35]. Thus, the current study hypothesizes that high RCSE will indirectly increase both heavy episodic drinking levels and alcohol-related problems through more stress and in turn more IC.

### 1.4. Error Management Theory—Cisgender Differences

Error Management Theory (EMT) suggests that natural selection has designed psychological mechanisms to regulate, and potentially bias, judgments and behavior in a way that minimizes costs [36]. Women have evolved to be the “choosier sex” when selecting a mating partner, as there is a greater investment of resources in offspring [37,38]. It has been demonstrated that women tend to under-infer men’s commitment as an evolutionary protective factor to avoid costs associated with single parenthood (e.g., increased investment of resources, lower mate value status, and reputational damage) [36,38]. Women’s selectiveness over mating partners may suggest that women will want to retain their mates in order to continue receiving benefits from the mates they chose. Subsequently, women engage in more mate retention behaviors (i.e., behaviors used to prevent the infidelity or defection of a partner) when their partners have higher incomes and have higher levels of status striving [39]. People who have high RCSE have been found to protect their relationships by engaging in mate retention behaviors [40]. In fact, “individuals with high levels of RCSE may be so desperate to maintain their relationships that they are willing to incur the risks that accompany the use of cost inflicting mate retention behaviors” [40] (p. 16). EMT suggests that women may be more likely to develop RCSE, thus the current study hypothesizes that women with high RCSE will experience more stress and therefore more IC along the distinct heavy episodic drinking and alcohol-related problem pathways compared to men.

### 1.5. Objectives and Hypotheses

In this study, we sought to determine if there was a relationship among RCSE, stress, IC, and alcohol-related problems. Informed by previous literature, we hypothesized a direct association between RCSE and stress [8,13,14]. Secondly, we hypothesized that stress will be directly linked with IC, heavy episodic drinking, and alcohol-related problems [41]. We expected our findings to be consistent with the ADM, such that alcohol dampens stress [9,10]. We predicted that greater levels of RCSE will be linked to greater stress, which, in turn, will be indirectly associated with greater IC along the alcohol-related problem pathway. That is, we anticipated that RCSE would be indirectly linked to more alcohol-related problems through the mediators of more stress and more IC. In addition, we expected our results to be consistent with the EMT [36]. Based on findings with RCSE, we predicted women will experience more stress in the context of RCSE than men in the sample. For cisgender women and men, we predicted that increased IC will be associated with more heavy episodic drinking and alcohol-related problems.

## 2. Methods

### 2.1. Participant Recruitment

College students were recruited via the SONA systems online survey tool. All recruited participants received class credit for participation in the current cross-sectional study. A total of 479 participants were recruited to participate in the current cross-sectional survey. The IRB’s approval and informed consent were obtained. We collected the data with paper and pencil surveys in person prior to COVID-19 pandemic events.

### 2.2. Measures

#### 2.2.1. The Relationship-Contingent Self-Esteem (RCSE) Scale [4] Included 11 Items about Thoughts and Behaviors in Committed Relationships

Example items of the RCSE scale are as follows: ‘‘My feelings of self-worth are based on how well things are going in my relationship,’’ and ‘‘When my partner and I fight, I feel bad about myself in general’’. Responses were rated on a 5-point Likert-type scale (1 = not at all like me and 5 = very much like me). Higher scores indicated one’s self-worth was more closely tied to one’s romantic relationship. The α for the RCSE scale was 0.85.

#### 2.2.2. Perceived Stress Scale (PSS) Consisted of 10 Items Which Measured the Degree to Which the Events in Life Are Stressful [42]

Items were on a 0–4 Likert scale (0 = never, 1 = almost never, 2 = sometimes, 3 = fairly often, and 4 = very often). Example items are “In the past month, how often have you been upset because of something that happened unexpectedly?” and “In the last month, how often have you felt difficulties were piling up so high that you could not overcome them?”. The α for the PSS was 0.81.

#### 2.2.3. Impaired Control over Drinking Scale Part III (ICS) [25] Included 10 Items

The ICS assessed perceived lack of control over drinking (e.g., an inability to stop drinking at will) using a 1–5 Likert scale format (1 = strongly disagree to 5 = strongly agree). Sample items included “I would have difficulty limiting the amount I drink” and “I would start to drink, even if I’d decided not to”. The α for the ICS was 0.81.

#### 2.2.4. Heavy Episodic Drinking [28] Included 1 Item and Measures Occasions of Heavy Episodic Drinking

The item stated “How many times in the past year did you drink 5 (please use 4 or more for women) or more bottles or cans of beer, glasses of wine, or drinks of distilled spirits on a single occasion?”. Responses were on an 8-point Likert scale (0 = never to 7 = daily or nearly daily).

#### 2.2.5. Problems with Alcohol Use [32]

These 12 items assessed moderate problems with alcohol use that may be indicative of alcohol use or dependence [32]. The items were assessed on a scale from 0 (never) to 3 (many times). Some sample items from this scale include using social occasions as an excuse to drink, depression after drinking, sneaking drinks or hiding bottles, and binge drinking. One impaired control (ICS) item was dropped, leaving eleven total items. The α for the Problems with Alcohol Use Scale 11-item measure was 0.86.

### 2.3. Statistical Approach

We calculated mean differences among the variables in our model with MANOVA command in Spss version 27.0. Using Mplus 8.3 [43], a structural equation model was evaluated with chi-square statistics, Root Mean Square Error of Approximation (RMSEA) [44,45], Comparative Fit Index (CFI), and Tucker–Lewis Index (TLI) [46]. Acceptable model fit was determined via RMSEA values ≤ 0.08, CFI values ≥ 0.95, and TLI values ≥ 0.90 [45,46,47]. Mediational effects were examined utilizing the parametric bootstrapped (k = 20,000) 90–99% asymmetric confidence interval technique for the estimates of the indirect effects (i.e., zero is not found in the interval of a mediated effect) [48,49]. In summary, a structural equation model evaluated relationships between RCSE, stress, IC, heavy episodic drinking, and alcohol-related problems.

## 3. Results

### 3.1. Participant Demographics

The sample consisted of 479 social drinkers (i.e., individuals who not yet been diagnosed with AUDs: 193 women and 286 men) who were 18 years of age or older from a large southwestern university located in the U.S.A (Table 1). The sample was 40% female, with a mean age of 19.91 years (*SD* = 2.82). Most of the sample was Caucasian (69%). The remainder of the sample included Hispanic (15%), Asian (7%), Black/African American (4%), American Indian/Alaskan Native (2%), or “other” (3%).

### 3.2. Cisgender Mean Differences in the Variables in the Model

**Figure 1:**
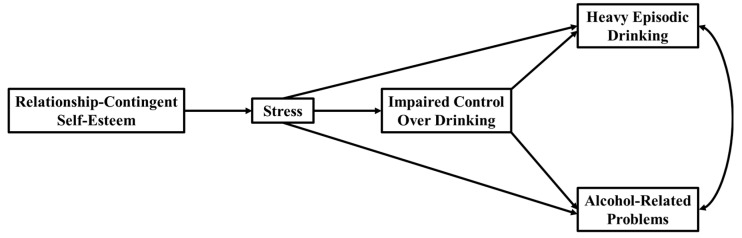
Conceptual model of all examined paths among the different variables in the model.

Table 2 presents the means, standard deviations, and correlations among all the variables in our conceptual model (Figure 1). There was not a significant difference for RCSE among men and women: F (1, 475) = 0.73, *p* = 0.393. Women had significantly higher levels of stress than men: F(1, 475) = 4.74, *p* = 0.03. Men had significantly higher levels of IC than women: F(1, 475) = 4.36, *p* = 0.037. Men had significantly higher levels of heavy episodic drinking than women: F(1, 27.15, *p* < 0.001. There was not a significant difference among men and women for our moderate alcohol-related problems measure: F(1, 475) = 0.39, *p* = 0.535.

### 3.3. Model Fit

Our model fit the data well with χ² (9 df) = 6.538, *p* = 0.3656; RMSEA = 0.019; 90% CI [0.000, 0.088]; CFI = 0.999; and TLI = 0.996. As our overall structural invariance test did exceed the critical value at *p* < 0.001 when all paths were constrained to equality [χ^2^(13df) = 30.881 minus χ^2^(6df) = 6.538 = χ^2^Δ (7 df) = 24.343, *p* < 0.001], it suggested that men and women do need to be modeled separately. Our hypothesis that the relationship from RCSE to stress would be stronger for women than men while in the correct direction was not supported with a one df structural invariant test of this specific path presented in Table 3. This means that the standardized beta of 0.52 with a Z score of 6.73 found in women is not significantly different from a standardized beta of 0.41 with a Z score of 4.74 found for men. Nevertheless, our model accounted for β = 0.154 (S.E.= 0.047; Z = 3.307, *p* < 0.001) of the variance in stress for women and only β = 0.066 (S.E, 0.040; Z = 1.630, *p* = 0.103) for men.

### 3.4. Direct Effects among Women

**Figure 2:**
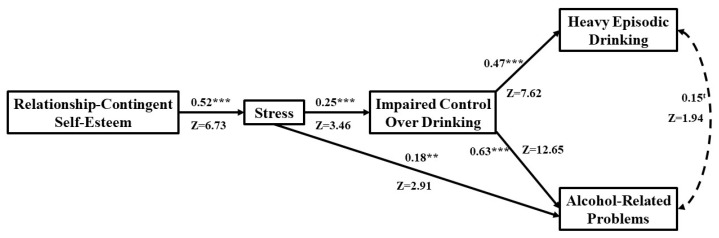
Fit path model for women, N = 193. Standardized coefficients are shown for women: t = trend at *p* = 0.052; ** *p* < 0.01; *** *p* < 0.001.

Higher levels of RCSE were directly linked to more stress among women (β = 0.52, Z = 6.73, *p* < 0.001; Figure 2). In addition, higher levels of stress were directly linked to more IC (β = 0.25, Z = 3.46, *p* < 0.001) and more alcohol-related problems among women (β = 0.18, Z = 2.91, *p* < 0.01). However, higher levels of stress were not directly linked to more heavy episodic drinking among women (β = −0.005; Z = -0.085; *p* = 0.932). Please note that while this direct link is not significant, the indirect path from stress to heavy episodic drinking is mediated by IC. For women, IC was directly linked to more heavy episodic drinking (β = 0.47, Z = 7.62, *p* < 0.001) and alcohol-related problems (β = 0.63, Z = 12.65, *p* < 0.001). As expected, heavy episodic drinking was directly associated with more alcohol-related problems among women (β = 0.15, Z =1.94, *p* = 0.052; trend), but only at the trend level.

### 3.5. Key Mediated Effects among Women

#### 3.5.1. Impaired Control and Heavy Episodic Drinking

Higher levels of RCSE were indirectly linked to more IC through more stress (indirect effect = 0.083; CI 95% [0.031, 0.145]) among women. In addition, higher levels of RCSE were indirectly linked to more heavy episodic drinking through more stress and IC (indirect effect = 0.094; CI 95% [0.033, 0.173]) among women. Further, higher levels of stress were indirectly linked to more heavy episodic drinking through increased IC (indirect effect = 0.300; CI 95% [0.117, 0.511] among women.

#### 3.5.2. Alcohol-Related Problems

Higher levels of RCSE were indirectly linked to more alcohol-related problems through more stress and IC (indirect effect = 0.047; CI 95% [0.017, 0.085]) among women. In addition, higher levels of stress were indirectly linked to more alcohol-related problems through more IC (indirect effect = 0.150; CI 95% [0.059, 0.254]) among women.

### 3.6. Direct Effects among Men

**Figure 3:**
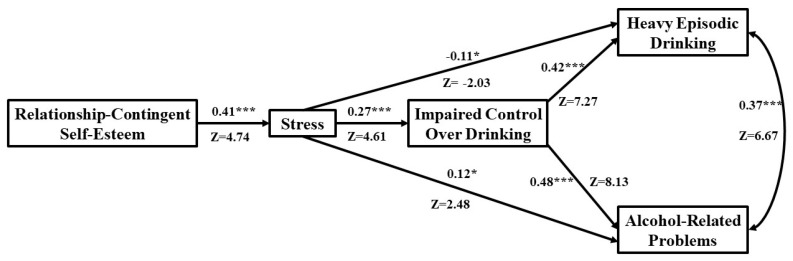
Fit path model for men, N = 286. Standardized coefficients are shown for men: * *p* < 0.05; *** *p* < 0.001.

Higher levels of RCSE were directly linked to more stress among men (β = 0.41, Z = 4.74, *p* < 0.001; Figure 3). In turn, higher levels of stress were directly linked to less heavy episodic drinking (β = −0.11, Z = −2.03, *p* < 0.05), but more IC (β = 0.27, Z = 4.61, *p* < 0.001) and more alcohol-related problems. More IC was also directly linked to more heavy episodic drinking (β = 0.42, Z = 7.27, *p* < 0.001) and alcohol-related problems among men (β = 0.48, Z = 8.13, *p* < 0.001). Finally, higher levels of heavy episodic drinking were directly associated with more alcohol related problems among men (β = 0.37, Z = 6.67, *p* < 0.001).

### 3.7. Key Mediated Effects among Men

#### 3.7.1. Impaired Control and Heavy Episodic Drinking

Higher levels of RCSE were positively indirectly linked to more IC through more stress (indirect effect = 0.079; CI 95% [0.033, 0.141]) among men. In addition, higher levels of RCSE were indirectly linked to more heavy episodic drinking through more stress and IC (indirect effect = 0.076; CI 95% [0.03, 0.143]) among men. Furthermore, higher levels of stress were indirectly linked to more heavy episodic drinking through more IC (indirect effect = 0.30; CI 95% [0.148, 0.487]) among men.

#### 3.7.2. Alcohol-Related Problems

Higher levels of stress were indirectly linked to more alcohol-related problems through more IC (indirect effect = 0.105; CI 95% [0.053, 0.170]) among men. Lastly, higher levels of RCSE were indirectly linked to more alcohol-related problems through more stress and IC (indirect effect = 0.027; CI 95% [0.01, 0.05]) among men.

## 4. Discussion

The Appraisal-Disruption Model (ADM) suggests that individuals use alcohol to dampen negative self-talk with one’s poor self-concept and self-worth [9,10]. This model presumes that individuals will experience heavy stress because of the way they evaluate themselves, which is dependent upon how an individual feels other people perceive them. Our findings are highly consistent with the ADM model. Our study indicates that higher RCSE is directly associated with higher levels of stress for both cisgender women and men. This is consistent with the extant literature, where RCSE has been associated with attachment anxiety (r = 0.26), general self-esteem (r = −0.29), and negative emotions (r = 0.38) [4] and interacts with lower relationship satisfaction to predict stronger coping motives for drinking among men [8]. The Error Management Theory (EMT) suggested that cisgender women’s stress levels would be impacted by higher RCSE more than men [36]. The hypothesis using evolutionary theory that women’s stress level would be impacted by higher RCSE more than man was unsupported and, thus, did not suggest structural invariance regarding the sexes at this pathway. Interestingly we found that RCSE was directly linked to more stress for both cisgender women and men. However, consistent with EMT the current model found that RCSE accounts for more variance among women regarding alcohol-related problems. In all, our present findings advocate that individuals with higher RCSE experience more stressful lives, suggesting a good target for therapeutic interventions, especially for women.

### 4.1. Impaired Control to Stress Link

Consistent with the extant literature [34,35], stress was directly linked to IC for both cisgender women and men. Nevertheless, our results are novel because our model illustrates that excess stress from those experiencing high RCSE can reduce an individual’s ability to intentionally limit their drinking. Consistent with the extant literature, IC was directly linked to heavy episodic drinking [29] and alcohol-related problems for both men and women [27,30,31]. Our findings suggest that RCSE indirectly impacts multiple drinking outcomes (i.e., IC, heavy episodic drinking, and alcohol-related problems) through the mediating mechanism of stress.

In our model, concerning RCSE, there was a stronger direct link between IC and alcohol-related problems for women than for men (women Z = 12.65, men Z = 8.13). Thus, college-aged women are more likely to experience alcohol-related problems following increased IC than men, particularly if indirectly influenced by higher RCSE. Our results expand upon existing literature [8] by demonstrating that RCSE is an important indirect predictor of alcohol-related problems, especially among women [13]. Nevertheless, consistent with existing literature [50,51] our model suggests there are stronger associations between heavy episodic drinking and alcohol-related problems among men than among women.

Our findings here are inconsistent with what might be predicted by the ADM [9,10], as we expected a direct link from stress to heavy episodic drinking. However, we did not find a direct link from stress to heavy episodic drinking among women, and the link was indeed negative among men. While this finding is inconsistent with the ADM, it is consistent with several studies showing internalizing symptomatology that is directly linked with alcohol-related problems but not directly linked to increased heavy episodic drinking and/or alcohol use quantity/frequency variables. For example, our findings are consistent with models of anxiety sensitivity [52], depression [53,54], neuroticism [55], and perfectionism discrepancy [56]. All these aforementioned studies reported direct links from internalizing symptoms to alcohol-related problems but not significant direct links to either heavy episodic or quantity/frequency alcohol use measures.

### 4.2. Cisgender Differences

Error Management Theory (EMT) suggests that women under-infer male commitment to minimize costs given uncertainty (i.e., unwanted pregnancy and single parenthood) [37]. Consistent with EMT, our current findings suggest that women who experience capricious self-esteem (i.e., RCSE) may also engage in costly decision making, such as drinking alcohol for stress relief [39]. Our model is consistent with EMT, as it explained more variance for women’s alcohol-related problems (R^2^ = 0.479) than for men (R^2^ = 0.280); this finding suggests that women’s drinking outcomes are more susceptible to relationship-contingent self-esteem (RCSE) than are men.

Our hypothesis that heavy episodic drinking would be significantly associated with alcohol-related problems was only partially supported. We found that the association between heavy episodic drinking and alcohol-related problems was structurally invariant among men and women. The finding that men typically exhibit a stronger association between heavy episodic drinking and alcohol-related problems is consistent with prior research [57,58].

### 4.3. Limitations and Future Directions

Although the results from the current study are novel, they must be taken into consideration within the limitations of the cross-sectional survey design among college students. First, the nature of this study is observational, thus there is no justification for casual relationships in the current model. Further, future research should elucidate the causal relationships between stress, dysregulated drinking, and alcohol-related outcomes [59,60]. Future studies should re-examine these links over time. This may provide insight into how an individual’s RCSE changes over time, and what implications this change may have on an individual’s quality of life. The current sample size was relatively small compared to other cross-sectional studies with the college student population [29,61]. Regarding generalizability, it would be interesting to see how RCSE in college students compares to other members of the community. Future studies should also implement other measurements of RCSE, such as daily diary reports from partners and other family members as a compliment to self-reports [4,14]. However, a strength of the current study is the relatively balanced proportion of cisgender women and men, since college student samples tend to oversample women [4,14]. Furthermore, the current study did not address the very important variable of attention deficit hyperactivity disorder (ADHD), which has been recently associated with IC. For instance, one article discusses that the underlying disinhibition in ADHD potentially mediates risk for potential adolescent substance use [62]. Moreover, another article found that children with ADHD are more likely to experience IC during young adulthood compared to children without ADHD [63]. Thus, future studies examining relationships between RCSE, IC, and drinking may also wish to examine ADHD. Moreover, future studies may wish to use an alcohol quantity/frequency outcome variable rather than heavy episodic drinking due to the possibility of some inflation of the association to alcohol problems due to a single binge drinking item as part of that measure. Lastly, one may need to replicate these findings in ethnically distinct groups, such as those coming from more collectivistic versus individualistic cultures.

Regardless of these limitations, this work remains a novel contribution to the literature. We are the first to demonstrate that RCSE is indirectly linked to heavy episodic drinking and alcohol-related problems through the mediating mechanisms of both stress and IC. This study is the first to show that RCSE is directly and strongly linked to stress for both cisgender women and men. We are also the first to demonstrate that RCSE has a stronger indirect influence on the drinking outcomes of women than for men. RCSE is simply an understudied construct regarding the etiology of drinking and is particularly important for cisgender women.

### 4.4. Clinical Implications

Tempering contingencies of self-worth are targets of therapeutic intervention [64]. Our current study found that individuals with high RCSE could be at increased risk of alcohol-related problems, especially women, due to increased stress and IC. Therapeutically, it may be useful to employ training promoting non-contingent self-esteem as well as emotional grounding self-efficacy [65]. Other important techniques include motivational interviewing or cognitive behavioral therapy. In both strategies, clinicians can assist individuals in understanding the motivation for relationship goals as well as coping skills when relationships are no longer flourishing [66,67]. Our findings suggest that reducing the stress associated with RCSE may reduce the formation of AUDs.

## Figures and Tables

**Table 1:** Demographic variables for all participants.

Demographic Variable	N	Percentage	*Mean*	*SD*
Age	479		19.91	2.82
Cisgender/biological sex				
Male	286	60		
Female	193	40		
Race/Ethnicity				
Caucasian		69		
Hispanic		15		
Asian		7		
Black/African American		4		
American Indian/Alaskan Native		2		
Other		3		

Note—based on a sample of 479 female and male college students.

**Table 2:** Means, standard deviations, and correlations among all variables in the model.

*M*	*SD*	Measure	1	2	3	4	5
**3.41**	**(0.60)**	1. Relationship-contingent self-esteem	1.00	**0.39**	**0.13**	**−0.01**	**0.22**
*3.45*	*(0.61)*						
**2.65**	**(0.64)**	2. Stress	*0.26*	1.00	**0.25**	**0.11**	**0.31**
*2.52*	*(0.69)*						
**1.71**	**(1.54)**	3. Impaired control over alcohol	*0.00*	*0.27*	1.00	**0.47**	**0.67**
*1.82*	*(1.60)*						
**1.68**	**(0.57)**	4. Heavy episodic drinking	*−0.01*	*0.00*	*0.39*	1.00	**0.41**
*2.45*	*(0.49)*						
**0.68**	**(0.75)**	5. Alcohol-related problems	*0.00*	*0.25*	*0.52*	*0.48*	1.00
*0.71*	*(0.63)*						

Note—based on a sample of 193 female (values on the upper diagonal in **bold**) and 286 male (values on the lower diagonal in *italics*). Range of possible scores for each measure: stress (1.20, 4.50); IC (1, 3.60); heavy episodic drinking (0, 5); alcohol-related problems (0, 2.640); RCSE (1, 5).

**Table 3:** Cisgender differences in the path coefficients.

Model	*x* ^2^	
Base Model (df = 6)	6.583	∆*x*^2^
Relationship-contingent self-esteem to stress	7.124	<1.000
Stress to impaired control	7.124	<1.000
Impaired control to heavy episodic drinking	7.171	<1.000
Stress to heavy episodic drinking	8.062	1.479
Impaired control to alcohol-related problems	19.822	13.284 ***
Stress to alcohol-related problems	7.739	1.156
Heavy episodic drinking with alcohol-related problems	12.299	5.761 *

Note—we examined structural invariance among cisgender women and men by looking at each path in the model with a 1 df test. The pathway from IC to alcohol-related problems is stronger for women than for men. The association between heavy episodic drinking to alcohol-related problems is stronger for men than for women. * *p* < 0.05; *** *p* < 0.001.

## Data Availability

Data are available upon request to the corresponding author.

## References

[1] Meyer S. (2008). 3—The End. New Moon.

[2] Conroy-Beam D., Goetz C.D., Buss D.M. (2016). What predicts romantic relationship satisfaction and mate retention intensity: Mate preference fulfillment or mate value discrepancies?. Evol. Hum. Behav..

[3] Salmon C. (2015). Parental investment and parent-offspring conflict. The Handbook of Evolutionary Psychology.

[4] Knee C.R., Canevello A., Bush A.L., Cook A. (2008). Relationship-contingent self-esteem and the ups and downs of romantic relationships. J. Personal. Soc. Psychol..

[5] Park L.E., Sanchez D.T., Brynildsen K. (2011). Maladaptive responses to relationship dissolution: The role of relationship contingent self-worth. J. Appl. Soc. Psychol..

[6] Crocker J., Park L.E. (2004). The costly pursuit of self-esteem. Psychol. Bull..

[7] Sanchez D.T., Good J.J., Kwang T., Saltzman E. (2008). When finding a mate feels urgent: Why relationship contingency predicts men’s and women’s body shame. Soc. Psychol..

[8] Rodriguez L.M., Knee C.R., Neighbors C. (2014). Relationships can drive some to drink: Relationship-contingent self-esteem and drinking problems. J. Soc. Pers. Relatsh..

[9] Sayette M.A. (1993). An appraisal-disruption model of alcohol’s effects on stress responses in social drinkers. Psychol. Bull..

[10] Sayette M.A. (1999). Does drinking reduce stress?. Alcohol Res. Health.

[11] Sayette M.A. (1994). Research Note Effects of Alcohol on Self-Appraisal. Int. J. Addict..

[12] Sinha R. (2007). The role of stress in addiction relapse. Curr. Psychiatry Rep..

[13] Tomaka J., Morales-Monks S., Shamaley A.G. (2013). Stress and coping mediate relationships between contingent and global self-esteem and alcohol-related problems among college drinkers. Stress Health.

[14] DiBello A.M., Rodriguez L.M., Hadden B.W., Neighbors C. (2015). The green eyed monster in the bottle: Relationship contingent self-esteem, romantic jealousy, and alcohol-related problems. Addict. Behav..

[15] Martens M.P., Neighbors C., Lewis M.A., Lee C.M., Oster-Aaland L., Larimer M.E. (2008). The roles of negative affect and coping motives in the relationship between alcohol use and alcohol-related-problems among college students. J. Stud. Alcohol Drugs.

[16] Capasso A., Jones A.M., Ali S.H., Foreman J., Tozan Y., DiClemente R.J. (2021). Increased alcohol use during the COVID-19 pandemic: The effect of mental health and age in a cross-sectional sample of social media users in the US. Prev. Med..

[17] Grossman E.R., Benjamin-Neelon S.E., Sonnenschein S. (2020). Alcohol consumption during the COVID-19 pandemic: A cross-sectional survey of US adults. Int. J. Environ. Res. Public Health.

[18] Rodriguez L.M., Litt D.M., Stewart S.H. (2020). Drinking to cope with the pandemic: The unique associations of COVID-19-related perceived threat and psychological distress to drinking behaviors in American men and women. Addict. Behav..

[19] American Psychological Association (2021). Stress in America: One Year Later, a New Wave of Pandemic Health Concerns.

[20] Sher K.J., Grekin E.R., Gross J.J. (2007). Alcohol and Affect Regulation. Handbook of Emotion Regulation.

[21] Mickelson K.D., Kessler R.C., Shaver P.R. (1997). Adult attachment in a nationally representative sample. J. Personal. Soc. Psychol..

[22] Levitt A., Cooper M.L. (2010). Daily alcohol use and romantic relationship functioning: Evidence of bidirectional, gender-, and context-specific effects. Personal. Soc. Psychol. Bull..

[23] DeHart T., Tennen H., Armeli S., Todd M., Affleck G. (2008). Drinking to regulate negative romantic relationship interactions: The moderating role of self-esteem. J. Exp. Soc. Psychol..

[24] Backer-Fulghum L.M., Patock-Peckham J.A., King K.M., Roufa L., Hagen L. (2012). The stress-response dampening hypothesis: How self-esteem and stress act as mechanisms between negative parental bonds and alcohol-related-problems in emerging adulthood. Addict. Behav..

[25] Heather N., Tebbutt J.S., Mattick R.P., Zamir R. (1993). Development of a scale for measuring impaired control over alcohol consumption: A preliminary report. J. Stud. Alcohol.

[26] Leeman R.F., Patock-Peckham J.A., Potenza M.N. (2012). Impaired control over alcohol use: An under-addressed risk factor for problem drinking in young adults?. Exp. Clin. Psychopharmacol..

[27] Patock-Peckham J.A., Morgan-Lopez A.A. (2006). College drinking behaviors: Mediational links between parenting styles, impulse control, and alcohol-related outcomes. Psychol. Addict. Behav..

[28] Wood M.D., Nagoshi C.T., Dennis D.A. (1992). Alcohol norms and expectations as predictors of alcohol use and problems in a college student sample. Am. J. Drug Alcohol Abus..

[29] Canning J.R., Patock-Peckham J.A., Walters K.J., Bauman D.C., Frohe T., Leeman R.F. (2020). Perfectionism discrepancy and falling short of the ideal self: Investigating drinking motives and impaired control on the road to alcohol-related problems. Personal. Individ. Differ..

[30] Patock-Peckham J.A., Cheong J., Balhorn M.E., Nagoshi C.T. (2001). A social learning perspective: A model of parenting styles, self-regulation, perceived drinking control, and alcohol use and problems. Alcohol. Clin. Exp. Res..

[31] Patock-Peckham J.A., King K.M., Morgan-Lopez A.A., Ulloa E.C., Filson Moses J.M. (2011). Gender-specific mediational links between parenting styles, parental monitoring, impulsiveness, drinking control, and alcohol-related problems. J. Stud. Alcohol Drugs.

[32] Rhea S.A., Nagoshi C.T., Wilson J.R. (1993). Reliability of sibling reports on parental drinking behaviors. J. Stud. Alcohol.

[33] Naidu E.S., Patock-Peckham J.A., Ruof A., Bauman D.C., Banovich P., Frohe T., Leeman R.F. (2019). Narcissism and devaluing others: An exploration of impaired control over drinking as a mediating mechanism of alcohol-related problems. Personal. Individ. Differ..

[34] Berberian S., Patock-Peckham J.A., Guarino K., Gupta T., Sanabria F., Infurna F. (2022). Does loneliness before the age of twelve indirectly affect impaired control over drinking, alcohol use, and problems through perceived stress?. Addict. Behav. Rep..

[35] Patock-Peckham J.A., Corbin W.R. (2022). Impaired control over drinking predicts changes in alcohol-related consequences over and above alcohol use and facets of impulsivity. Addict. Behav..

[36] Haselton M.G., Buss D.M. (2000). Error management theory: A new perspective on biases in cross-sex mind reading. J. Personal. Soc. Psychol..

[37] Buss D.M., Schmitt D.P. (1993). Sexual strategies theory: An evolutionary perspective on human mating. Psychol. Rev..

[38] Trivers R. (1972). Parental Investment and Sexual Selection.

[39] Buss D.M., Shackelford T.K. (1997). From vigilance to violence: Mate retention tactics in married couples. J. Personal. Soc. Psychol..

[40] Holden C.J., Zeigler-Hill V., Shackelford T.K., Welling L.L. (2018). The impact of relationship-contingent self-esteem on mate retention and reactions to threat. Pers. Relatsh..

[41] Muniz F.B., Bradfield A.R., Belton D.A., Canning J.R., Dergal S., Esquer A.M., Patock-Peckham J.A. (2016). The indirect influences of authoritative parenting on alcohol problems through the mediating mechanisms of stress and impaired control over drinking. Alcohol. Clin. Exp. Res..

[42] Cohen S., Kamarck T., Mermelstein R. (1994). Perceived stress scale. Meas. Stress Guide Health Soc. Sci..

[43] Muthén L.K. (2010). Mplus User’s Guide.

[44] Browne M.W., Cudeck R. (1992). Alternative ways of assessing model fit. Sociol. Methods Res..

[45] Hu L.T., Bentler P.M. (1998). Fit indices in covariance structure modeling: Sensitivity to under-parameterized model misspecification. Psychol. Methods.

[46] Tucker L.R., Lewis C. (1973). A reliability coefficient for maximum likelihood factor analysis. Psychometrika.

[47] Bentler P.M. (1990). Comparative fit indexes in structural models. Psychol. Bull..

[48] Hancock G.R., Liu M. (2012). Bootstrapping standard errors and data-model fit statistics in structural equation modeling. Handbook of Structural Equation Modeling.

[49] MacKinnon D.P. (2008). Introduction to Statistical Mediation Analysis.

[50] Pitkänen T., Lyyra A.L., Pulkkinen L. (2005). Age of onset of drinking and the use of alcohol in adulthood: A follow-up study from age 8–42 for females and males. Addiction.

[51] Wechsler H., Nelson T.F. (2001). Binge drinking and the American college students: What’s five drinks?. Psychol. Addict. Behav..

[52] Ebbert A.M., Patock-Peckham J.A., Luk J.W., Voorhies K., Warner O., Leeman R.F. (2018). The mediating role of anxiety sensitivity in uncontrolled drinking: A look at gender-specific parental influences. Alcohol. Clin. Exp. Res..

[53] Patock-Peckham J.A., Morgan-Lopez A.A. (2007). College drinking behaviors: Mediational links between parenting styles, parental bonds, depression, and alcohol problems. Psychol. Addict. Behav..

[54] Patock-Peckham J.A., Morgan-Lopez A.A. (2009). Mediational links among parenting styles, perceptions of parental confidence, self-esteem, and depression on alcohol-related problems in emerging adulthood. J. Stud. Alcohol Drugs.

[55] Patock-Peckham J.A., Morgan-Lopez A.A. (2009). The gender specific mediational pathways between parenting styles, neuroticism, pathological reasons for drinking, and alcohol-related problems in emerging adulthood. Addict. Behav..

[56] Patock-Peckham J.A., Corbin W.R. (2019). Perfectionism and self-medication as mediators of the links between parenting styles and drinking outcomes. Addict. Behav. Rep..

[57] Dawson D.A., Grant B., Stinson F.S., Chou P.S. (2004). Another look at heavy episodic drinking and alcohol use disorders among college and non-college youth. J. Stud. Alcohol.

[58] Jackson K.M. (2008). Heavy Episodic Drinking: Determining the predictive utility of 5 or more drinks. Psychol. Addict. Behav..

[59] Li Y., Ramoz N., Derrington E., Dreher J.C. (2020). Hormonal responses in gambling versus alcohol abuse: A review of human studies. Prog. Neuro-Psychopharmacol. Biol. Psychiatry.

[60] Uhart M., Wand G.S. (2009). Stress, alcohol and drug interaction: An update of human research. Addict. Biol..

[61] Frohe T., Leeman R.F., Cheong J., Belton D.A., Patock-Peckham J.A. (2020). Novel associations among trauma, mindfulness, and impaired control over alcohol use. Mindfulness.

[62] Fillmore M.T. (2012). Drug abuse and behavioral disinhibition. Drug Abuse and Addiction in Medical Illness: Causes, Consequences and Treatment.

[63] Wang F.L., Pedersen S.L., Kennedy T.M., Gnagy E.M., Pelham W.E., Molina B.S. (2021). Persistent attention-deficit/hyperactivity disorder predicts socially oriented, but not physical/physiologically oriented, alcohol problems in early adulthood. Alcohol. Clin. Exp. Res..

[64] Lakey C.E., Hirsch J.K., Nelson L.A., Nsamenang S.A. (2014). Effects of contingent self-esteem on depressive symptoms and suicidal behavior. Death Stud..

[65] Srikala B., Kumar K.K. (2010). Empowering adolescents with life skills education in schools—School mental health program: Does it work?. Indian J. Psychiatry.

[66] Hamarta E. (2009). A prediction of self-esteem and life satisfaction by social problem solving. Soc. Behav. Personal. Int. J..

[67] Waite P., McNanus F., Shafran R. (2012). Cognitive behaviour therapy for low self-esteem: A preliminary randomized controlled trial in a primary care setting. J. Behav. Ther. Exp. Psychiatry.

